# Educational effectiveness of introductory training for the Rapid Response System in Japan

**DOI:** 10.1186/cc12429

**Published:** 2013-03-19

**Authors:** T Kodama, M Nakagawa, E Kawamoto, S Fujiwara, H Imai, S Fujitani, K Atagi

**Affiliations:** 1 The University of Texas Southwestern Medical Center at Dallas, TX, USA; 2 Social Insurance Kinan Hospital, Tanabe-City, Japan; 3 Mie University Hospital, Tsu-City, Japan; 4 NHO Ureshino Medical Center, Ureshino-City, Japan; 5 Tokyobay UrayasuIchikawa Medical Center, Urayasu-City, Japan; 6 Osaka City General Hospital, Osaka-City, Japan

## Introduction

Interest in safety and clinical outcomes of inpatients has been growing in Japan, because the 100,000 Lives Campaign was introduced under the Japanese Patient Safety Act in 2008. In this act, an introduction of the Rapid Response System (RRS) was one of the mainstreams to inpatients' care. However, many Japanese healthcare providers cannot understand how to achieve the introduction of the RRS, because there are few who have knowledge of the system. Therefore, we developed a new introductory training course for the RRS. The educational effectiveness was analyzed through the surveillance questionnaires after the course.

## Methods

The educational program includes a lecture series concerning the outline and management methods, introduction of facilities that have already deployed, small group discussions, and teaching methods-of-training for the medical emergency team using a simulator. Evaluation was made in the five-point scale by 82 participants (58 physicians, 16 nurses and eight other professions) throughout seven courses. The questionnaires are: A. understanding of RRS, B. knowledge acquisition about patient safety, C. expectation for decreasing the cardiopulmonary arrest by RRS, and D. expectation for decreasing the psychological burden by RRS.

## Results

Seventy-three participants (89.0%) answered the questionnaires. The numbers of participants who scored more than four points were as follows: A. was 71 (97.2%), B. was 70 (95.9%), C. was 64 (87.7%), and D. was 68 (93.2%), respectively. The majority of participants obtained the correct knowledge, and had a solid understanding for the RRS. It was evident that providing abundant material and didactic lectures traced from the introduction to management, and collecting and resolving the questions, promoted comprehension. However, there is a limitation of whether or not the participants introduce the RRS into their own institutions. It is essential to improve the course and continue to support the activities of the participants.

## Conclusion

Our training course may promote the introduction and dissemination of the RRS in Japan.
